# Radar HRRP Sequence Target Recognition Based on a Lightweight Spatiotemporal Fusion Network

**DOI:** 10.3390/s26010334

**Published:** 2026-01-04

**Authors:** Xiang Li, Yitao Su, Xiaobin Zhao, Junjun Yin, Jian Yang

**Affiliations:** 1 Department of Electronic Engineering, Tsinghua University, Beijing 100084, China; 2 School of Computer and Communication Engineering, University of Science and Technology Beijing, Beijing 100083, China

**Keywords:** HRRP sequence recognition, lightweight spatiotemporal fusion, transform domain, adaptive focal loss

## Abstract

High-resolution range profile (HRRP) sequence recognition in radar automatic target recognition faces several practical challenges, including severe category imbalance, degradation of robustness under complex and variable operating conditions, and strict requirements for lightweight models suitable for real-time deployment on resource-limited platforms. To address these problems, this paper proposes a lightweight spatiotemporal fusion-based (LSTF) HRRP sequence target recognition method. First, a lightweight Transformer encoder based on group linear transformations (TGLT) is designed to effectively model temporal dynamics while significantly reducing parameter size and computation, making it suitable for edge-device applications. Second, a transform-domain spatial feature extraction network is introduced, combining the fractional Fourier transform with an enhanced squeeze-and-excitation fully convolutional network (FSCN). This design fully exploits multi-domain spatial information and enhances class separability by leveraging discriminative scattering-energy distributions at specific fractional orders. Finally, an adaptive focal loss with label smoothing (AFL-LS) is constructed to dynamically adjust class weights for improved performance on long-tail classes, while label smoothing alleviates overfitting and enhances generalization. Experiments on the MSTAR and CVDomes datasets demonstrate that the proposed method consistently outperforms existing baseline approaches across three representative scenarios.

## 1. Introduction

Radar technology has consistently played a pivotal role in both military and civilian domains. With the rapid development of modern radar technology, radar imaging technology has gone way beyond traditional detection limits. High-resolution techniques, primarily achieved through wideband signal transmission and pulse compression, enable the acquisition of detailed target signatures such as the high-resolution range profile (HRRP), making fine-grained target recognition by radar possible. Consequently, radar automatic target recognition (RATR) has become an important task in radar applications. The core principle of this technology involves extracting and analyzing features from radar measurements using advanced signal processing algorithms and pattern recognition methods to achieve automatic target classification and identification. HRRP, derived from wideband radar systems, represents the vector sum of target scattering echoes projected along the radar’s radial direction, containing crucial discriminative information such as target shapes, structural dimensions, and scattering center distributions [1]. HRRP data exhibit unique advantages due to their ease of acquisition, convenient processing, efficient storage, and rich structural information [2]. These distinct characteristics make HRRP an important technical support for RATR, offering reliable solutions for efficient and precise target identification.

From a methodological perspective, HRRP-based RATR methods can primarily be categorized into three groups: (1) feature extraction-based methods that identify robust features from HRRP signals and leverage designed classifiers for target differentiation, (2) statistical model-based methods that construct probability distribution models using training data and classify target categories by calculating the likelihood probabilities of test samples, and (3) deep learning-based methods that enable end-to-end recognition through neural networks for autonomous feature extraction [3].

In feature extraction-based methods, the recognition performance is critically influenced by the effectiveness and accuracy of feature selection. The core challenge lies in constructing feature spaces with translation invariance and intrinsic target separability, the design of which heavily relies on empirical expertise. Existing research has achieved preliminary progress: Studies [4,5,6] demonstrated feasibility in target recognition tasks by selecting physically meaningful features with strong representational capabilities from HRRP data. Meanwhile, through the integration of signal processing and pattern recognition theories, these methods have evolved into multidimensional frameworks incorporating innovative approaches such as time-frequency analysis [7,8], sparse representation [9,10], and nonlinear transformations [11,12]. Although such methods exhibit theoretical advantages in characterizing target scattering properties, significant technical limitations persist. Crucially, such approaches exhibit limited generalizability in multi-target recognition contexts, constrained by category-specific feature engineering and vulnerability to long-tailed distributions characterized by dominant majority classes and severely scarce minority categories that induce biased learning.

Statistical model-based HRRP target recognition methods characterize the distribution patterns of target scattering properties through probabilistic modeling. The core principle involves treating radar echoes as stochastic processes and establishing probabilistic mapping relationships between target types and echo data via statistical inference. Representative statistical models include adaptive Gaussian classifiers, Gamma mixture models, Gamma–Gaussian mixture models, and factor analysis (FA) models. Du et al. systematically developed statistical modeling approaches to address recognition robustness in noisy environments [13,14,15]. Notably, since the introduction of FA models into HRRP statistical recognition in 2008, significant research advancements have been achieved in this domain [16,17,18]. These methods fundamentally depend on prior distribution assumptions. This intrinsic constraint creates a theoretical mismatch with HRRP’s inherent non-Gaussian characteristics and multimodality, ultimately limiting complex scattering pattern characterization.

Deep learning approaches leverage HRRP sequences’ inherent spatiotemporal characteristics, where temporal dependencies from azimuth variations and spatial correlations across range cells provide complementary discriminative information. Initial efforts focused on temporal modeling through recurrent architectures, achieving notable success in aircraft target recognition [2,19,20]. While existing studies have achieved progress in target recognition through temporal feature modeling with recurrent neural networks, their frameworks exhibit notable limitations by neglecting spatial correlation characteristics within HRRP sequences. Consequently, the effective integration of spatiotemporal joint features in HRRP has emerged as a critical research focus in this field. Wan et al. [21] developed a hybrid recognition framework integrating convolutional neural networks (CNNs) with bidirectional recurrent neural networks (BiRNNs), where CNN modules extract spatial correlation features from HRRP data, while BiRNN modules capture temporal dependencies across range cells. Wang et al. [22] innovatively combined CNNs with Bidirectional Encoder Representations from Transformers (BERT), employing convolutional modules to characterize local spatial structures and utilizing BERT’s multi-head attention mechanisms to extract temporal features from HRRP sequences. Wu et al. [23] advanced this approach by constructing a fusion network that initially pre-extracts features through BERT, subsequently employs multi-scale CNNs and bidirectional gated recurrent networks to capture local characteristics and long-range dependencies, respectively, and finally concatenates features to integrate advantages from diverse networks. Establishing effective spatiotemporal feature extraction mechanisms to achieve complementary fusion of HRRP’s spatiotemporal characteristics and extending these to multi-target recognition scenarios remains a highly valuable research frontier.

With the rapid advancement of deep learning and growing academic interest in Transformer methodologies, recent years have witnessed increasing exploration of their applications in HRRP recognition. Zhang et al. [24] developed a feature-guided Transformer model that integrates manually designed features into attention modules to focus on HRRP range cells with rich scattering information, significantly improving recognition accuracy under small-sample conditions. Diao et al. [25] proposed a positional embedding-free CNN–Transformer hybrid architecture optimized for HRRP data characteristics, effectively eliminating the need for traditional positional encoding. Wang et al. [26] employed dual-branch Transformer encoders to separately extract temporal and spatial features, with designed attention fusion mechanisms achieving adaptive feature weighting. Gao et al. [27] innovatively introduced polarization preprocessing modules that combine artificial features with CNNs to enhance feature representation, constructing a Vision Transformer-based framework that substantially improves local and temporal feature extraction. Although current studies improve performance through module stacking, they commonly suffer from overfitting risks caused by excessive parameters. Insufficient emphasis on model lightweighting poses challenges in practical deployment on edge devices.

Current HRRP radar target recognition research primarily focuses on two typical data environments: (1) recognition methodologies under class-balanced conditions, primarily addressing cooperative target identification tasks. Under such conditions, controllable detection conditions enable researchers to acquire abundant high-quality samples, thereby facilitating the adoption of complex models like deep neural networks to leverage their powerful feature extraction capabilities for high-precision recognition. (2) Recognition technology exploration under data-limited and class-imbalanced conditions, mainly targeting non-cooperative target identification. Due to factors such as long detection ranges and complex electromagnetic environments, effective sample acquisition for such targets proves challenging, resulting in datasets exhibiting typical long-tailed distribution characteristics. It is noteworthy that in practical applications such as national defense and aerospace, the identification of non-cooperative targets with high observational value presents more urgent demands. These operational environments face three core challenges: First, target echoes are susceptible to noise interference, leading to significantly reduced signal-to-noise ratios; Second, there exists a prominent contradiction between target category diversity and limited training samples; Third, measured data inherently exhibit long-tailed distribution properties. Establishing effective recognition models under multi-category imbalanced data conditions has emerged as a critical scientific challenge in advancing the practical application of radar automatic recognition technology.

Researchers are actively exploring diverse technical approaches to address class imbalance issues in HRRP target recognition. Yin et al. [28] proposed an Adaptive Uniform Manifold Approximation and Projection (AUMAP) segmentation algorithm, which mitigates data imbalance by modifying the CNN loss function into a focal loss formulation. Jia et al. [29] developed a memory-based neural network (MBNN) for imbalanced data conditions: CNN-extracted features are processed through a memory module that records misclassified and low-confidence samples, followed by long short-term memory (LSTM) based fusion of classified samples with buffer-stored similar samples for final decision-making. Zhang et al. [30] introduced an open-set imbalanced recognition network integrating dual-attention mechanisms, memory modules, across functions, and decoupled training strategies, optimizing intra-class and inter-class similarity constraints via angular penalty loss. Guo et al. [31] established a transfer learning framework involving pretrained models with source domain data, subsequently resetting fully-connected and output layer parameters, and proposed a novel loss function to suppress inter-class bias for measured HRRP datasets with limited samples and class imbalance. Wu et al. [32] created a weighted synthetic minority oversampling technique (SMOTE) algorithm that dynamically allocates synthetic weights based on Euclidean distances among minority samples, combined with multi-scale CNN–Transformer encoder attention models to enhance multi-level feature classification accuracy. Tian et al. [33] designed a gradient-guided class re-balancing loss (GRB Loss) for space micro-motion targets, which dynamically assigns weights according to accumulated positive-negative gradient ratios at classification nodes, ensuring recognition robustness across varying imbalance ratios.

As a core technology of RATR, HRRP sequence-based target recognition plays a critical role in determining battlefield perception capabilities within complex environments. Nevertheless, existing methodologies exhibit significant technical limitations in feature synergy utilization, computational efficiency, and scenario generalization. Primarily, inadequate collaborative modeling of spatiotemporal features restricts cross-dataset adaptability. While HRRP sequences inherently contain temporal pose evolution characteristics and spatial scattering structural information, current approaches predominantly focus on temporal feature extraction via recurrent neural networks or spatial correlation mining through convolutional architectures, resulting in inadequate collaborative representation of spatiotemporal features. This deficiency leads to drastic performance degradation in complex battlefield environments involving variant targets or low signal-to-noise conditions, revealing fundamental weaknesses in generalization robustness. Secondly, structural redundancy in models elevates overfitting risks. Contemporary mainstream approaches predominantly adopt complex module stacking strategies, such as deep Transformer encoders and multi-branch convolutional architectures, to enhance recognition performance. This architectural complexity results in exponential escalation of both parameter volumes and computational demands. However, the inherent contradiction between embedded radar devices’ limited computing power and resource-intensive model requirements not only hinders deployment feasibility but also exacerbates overfitting risks due to insufficient training data, severely compromising practical utility. Finally, biased data distribution exacerbates generalization deterioration. Current research predominantly concentrates on class-balanced datasets, with insufficient investigation into multi-class long-tailed distribution challenges. Traditional cross-entropy loss functions, which apply uniform weighting to all samples, induce excessive model adaptation to majority classes while neglecting discriminative feature learning for high-value tail categories. Consequently, this imbalance triggers catastrophic performance degradation in critical minority class recognition.

To address the above technical challenges in HRRP sequence target recognition, this paper proposes a lightweight spatiotemporal fusion-based (LSTF) HRRP sequence target recognition method. The main innovations are summarized as follows:

1. **LSTF-based HRRP sequence target recognition framework:**
  We propose an LSTF HRRP sequence target recognition method with a dual-branch feature extraction architecture, in which a temporal feature encoding module and a spatial fully convolutional module are jointly fused to capture both target pose evolution and scattering structure distribution.

2. **Lightweight Transformer encoder for temporal modeling:**
  A Transformer encoder based on group linear transformations (TGLT) is designed to effectively model temporal dynamics in HRRP sequences while significantly reducing parameter count and computational complexity, enabling real-time deployment on edge devices.

3. **Transform-domain spatial feature extraction network:**
  We develop a transform-domain spatial feature extraction network that integrates the fractional Fourier transform (FrFT) with an enhanced squeeze-and-excitation fully convolutional network (FSCN). By exploiting multi-domain spatial representations, the proposed network enhances the discriminability of scattering energy distributions across target classes at specific fractional orders, leading to improved classification performance.

4. **Adaptive loss for long-tail recognition:**
  An adaptive focal loss with label smoothing (AFL-LS) is introduced to dynamically reweight imbalanced classes, improving recognition of long-tail categories. The incorporation of label smoothing further mitigates overfitting and significantly enhances the model’s generalization ability.

The remainder of this paper is organized as follows. Section 2 details the proposed LSTF network architecture, including the overall framework, the TGLT-based temporal feature encoder module, the transform-domain spatial feature fully convolutional module, and the decision fusion and recognition module. Section 3 introduces the proposed AFL-LS function, designed to address class imbalance. Section 4 presents the experimental results and analysis, including dataset descriptions, recognition performance comparisons, ablation studies, and feature visualizations. Finally, Section 5 concludes the paper.

Notably, the proposed method also exhibits strong potential for extension to target detection tasks in high-frequency surface wave radar (HFSWR) systems, particularly in high-resolution maritime surveillance scenarios. For ship detection and discrimination from sea clutter on range–Doppler maps, the lightweight TGLT-based encoder can effectively capture the temporal dynamics of ship echoes, while the transform-domain FSCN module enhances feature separability by exploiting scattering energy distributions in the fractional Fourier domain. Moreover, the AFL-LS loss mitigates the severe imbalance between sparse ship targets and dense sea clutter, thereby improving detection robustness. Benefiting from its lightweight architecture and low computational overhead, the proposed framework is well aligned with the real-time processing requirements of operational HFSWR systems. These characteristics indicate that the proposed method is not limited to HRRP sequence recognition but also holds broader practical value for cross-domain radar target analysis.

## 2. Method

### 2.1. Overall Network Architecture

**Figure 1:**
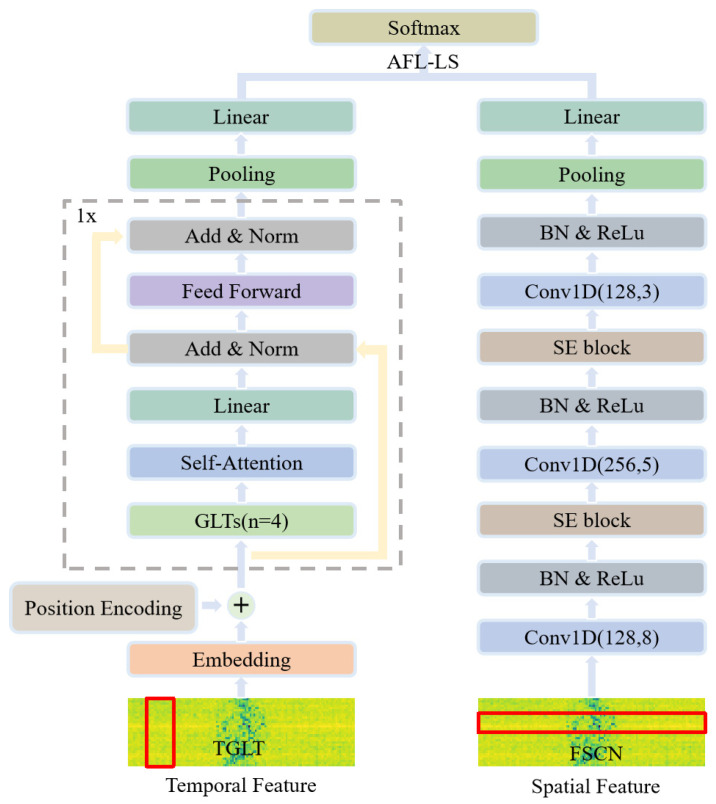
LSTF network structure.

The architecture of the proposed recognition method is shown in Figure 1, featuring a dual-branch structure with three main components: the temporal feature encoder, the spatial feature convolutional module, and the decision fusion module. In the temporal branch, the TGLT efficiently models temporal dynamics by capturing dependencies across different time steps through a self-attention mechanism, while reducing parameters and computational cost for real-time edge deployment. In the spatial branch, a transform-domain feature extraction network combining FrFT with an enhanced FSCN exploits multi-domain spatial information to enhance feature discriminability. Specifically, the FrFT projects the data into a joint time-frequency domain to enhance separability, and the subsequent FSCN (comprising Conv1D, SE blocks, and pooling) extracts multi-scale local structural features. Finally, the decision fusion module employs an AFL-LS to dynamically adjust class weights for improved long-tail recognition and applies label smoothing to boost generalization.

We assume the input HRRP sequence is $X\;={\;\left[x_{1}\;x_{2}\;\cdots \;x_{n}\right]}^{T}$, where the sequence has dimensions n × c, with n representing the length of the HRRP sequence and c representing the channel dimension of the HRRP sequence. For the temporal branch, a high-dimensional linear mapping is first applied to map the channel dimension of the sequence from c dimensions to $d_{\mathrm{model}}$ dimensions as

(1) $$Y_{\mathrm{token}}=XW_{e}$$

where $Y_{\mathrm{token}}$ denotes the sequence after linear transformation, with the channel dimension being $d_{\mathrm{model}}$, and $W_{e}\in R^{c\times d_{\mathrm{model}}}$ is the linear transformation matrix. Positional encoding is added to Y as

(2) $$Y=Y_{\mathrm{token}}+{\;X}_{p}$$

where $X_{p}\in R^{n\times d_{\mathrm{model}}}$ represents the positional encoding matrix. The temporal feature encoder then processes Y using a Transformer encoder to extract features, resulting in the temporal feature $O_{T}$. The spatial branch takes $X^{T}$ as input and extracts features through multiple stacked layers of one-dimensional convolution, batch normalization, squeeze-and-excitation, and pooling, ultimately yielding the spatial feature $O_{S}$.

The decision fusion recognition module uses $O_{T}$ and $O_{S}$ as inputs and performs classification using a fully connected layer and a Softmax function for each branch, thereby obtaining the classification results $P_{T}$ for the temporal branch and $P_{S}$ for the spatial branch. To achieve adaptive fusion of the temporal and spatial branches, we establish learnable decision weight matrices $W_{T}$ and $W_{S}$. The final recognition result P is computed as

(3) $$P\;=\mathrm{Softmax}\left({W_{T}P}_{T}+{W_{S}P}_{S}\right)$$

where $W_{T},\;W_{S}\in R^{1\times t}$ are the learnable decision weight matrices, and t represents the number of target categories. These matrices are model parameters optimized during training. Each vector contains t scalar weight values, corresponding to the t target classes. Their function is to perform an adaptive, category-specific fusion. The Softmax function is then used to constrain the output values of the final recognition result.

Weather factors such as rain, fog, and snow primarily affect HRRP data by reducing signal-to-noise ratio (SNR) and distorting scattering center features, and our proposed LSTF method addresses these challenges through its core designs: the TGLT-based lightweight Transformer encoder maintains stable temporal feature extraction under low SNR, the FrFT-integrated FSCN module decouples weather-induced noise from spatial structural information, and the AFL-LS loss function dynamically adjusts weights to focus on reliable features distorted by weather. Experimental results on low-SNR datasets (Dataset 3) show that the method achieves over 93% accuracy at 15 dB SNR (simulating light rain/fog) and 79.96% accuracy at 5 dB SNR (simulating heavy rain/snow), outperforming baselines by 4–7% under harsh conditions. Overall, the method exhibits robust adaptability across typical weather scenarios, maintaining high recognition performance in mild weather and retaining usability under extreme conditions, which is crucial for practical radar deployment.

### 2.2. Temporal Feature Encoder Module

**Figure 2:**
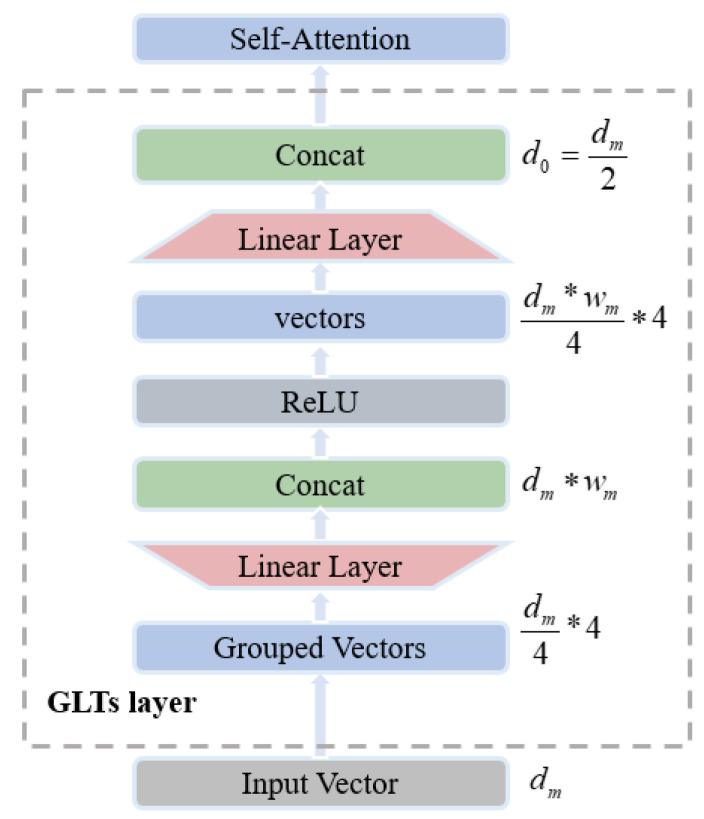
Illustration of the structure of the group linear transformations layer.

The encoder is a critical component of the Transformer architecture. For the standard Transformer encoder, the requirement to process global information from the input data results in high computational complexity, which leads to a large number of model parameters and extended training times. These characteristics are not conducive to deployment on edge devices. In the method proposed in this paper, we utilize group linear transformations (GLTs) to lighten the encoder, as illustrated in Figure 2. GLTs leverage the fact that HRRP sequences exhibit localized temporal dependencies and redundant interchannel correlations. By partitioning input channels into groups, GLTs introduce structured sparsity that aligns with the low-dimensional nature of radar scattering signatures. This design reduces model capacity while preserving key temporal dynamics, effectively mitigating overfitting with limited training data. From a matrix approximation perspective, GLTs act as a block-diagonal low-rank approximation of a full linear projection, retaining sufficient expressiveness for pose variations while significantly reducing computational complexity. This approach significantly reduces the computational load and training time by simplifying the feature transformation and attention operations within the encoder. Specifically, the temporal feature encoder is built with a group linear transformation layer that performs efficient dimension expansion and reduction, followed by a self-attention layer to capture long-range temporal dependencies. A dropout layer and residual connections are included to stabilize optimization and prevent overfitting, while a lightweight feed-forward network further decreases the parameter size through compressed intermediate dimensions. Finally, a pooling layer aggregates the temporal features into a compact representation for subsequent recognition.

The group linear transformation layer consists of two operations: dimensionality expansion and reduction. Initially, the input data are divided into multiple groups, each of which is mapped to a higher-dimensional space and expanded. By concatenating these vectors, high-dimensional features that are more discriminative are obtained. After processing these features with the rectified linear unit (ReLU) function, they are reduced in dimensionality within each group, and the resulting low-dimensional features are concatenated to serve as the input to the self-attention layer. This two-stage transformation effectively integrates features across different dimensions, yielding better performance than simple linear encoding. Consequently, replacing the multi-head attention mechanism in the standard Transformer with self-attention and reducing the input dimensionality to half that of the original significantly decreases the computational load.

The self-attention layer calculates the relevance of sequence data using query and key vectors to derive the attention matrix, which captures the global dependencies within the sequence. The resulting attention features are expressed as

(4) $$A=Attention(Q,K,V)=Soft\max(\frac{QK^{T}}{\sqrt{d_{k}}})V$$

where the query vector $Q\;=\;YW_{Q}$, the key vector $K\;=\;YW_{K}$, and the value vector $V\;=\;YW_{V}$ are defined, with $W_{Q},\;W_{K},\;W_{V}$ being learnable weight matrices. To maintain consistency between input and output dimensions and to prevent the loss of original features due to the depth of the model, the attention feature vector A undergoes dimensionality expansion and residual connection processing. The calculation for the feature F is given by

(5) $$F\;=W_{L}A+Y$$

where $W_{L}$ is the parameter matrix for dimensionality expansion of the attention feature A.

In the lightweight feed-forward neural network, the input dimensionality $d_{m}$ is first reduced to $\frac{d_{m}}{4}$ and then expanded back, with the parameter count reduced to 1/16 of the original while maintaining performance. The final features are computed as

(6) $$O_{T\;}=\mathrm{GAP}\left(F+\left(\left(\mathrm{ReLU}\;(FW_{1}+b_{1}\right)\right)W_{2}+{\;b}_{2}\right)$$

where $W_{1}$ and $b_{1}$ are the parameter matrix and bias vector for dimensionality reduction, and $W_{2}$ and $b_{2}$ are the parameter matrix and bias vector for dimensionality expansion, with $GAP\left(\cdot \right)$ denoting the global average pooling operation and $\mathrm{ReLU}\left(\cdot \right)$ representing the nonlinear activation function.

### 2.3. Transform Domain Spatial Feature Fully Convolutional Module

The local features of HRRP target sequences contain valuable information, such as the peaks and troughs of the echo signals, which are crucial for target recognition and classification. CNNs can effectively extract local features through convolution operations, thereby capturing the spatial structural information of HRRP data. The spatial feature fully convolutional module in the proposed method consists of one-dimensional convolutional layers, batch normalization (BN) layers, ReLU activation functions, squeeze-and-excitation (SE) blocks, and pooling layers.

**Figure 3:**
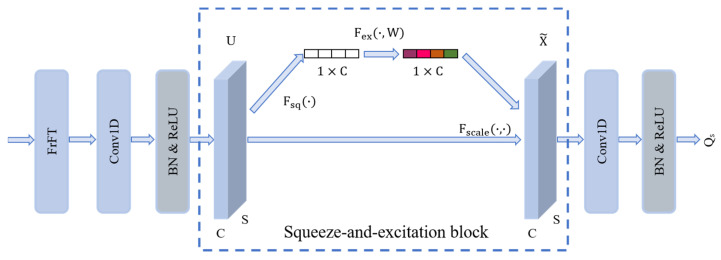
Squeeze-and-excitation block.

The convolutional blocks, composed of one-dimensional convolutional layers, batch normalization layers, and ReLU activation functions, are employed to extract spatial features from the input data. The spatial feature extraction module consists of three convolutional blocks with kernel sizes of 8, 5, and 3, respectively. These sizes enable the capture of spatial information at different scales, thereby enhancing the accuracy of target recognition. Following the first two convolutional blocks, SE blocks are incorporated, as illustrated in Figure 3. The SE blocks perform feature recalibration using adaptive average pooling and fully connected layers, leveraging global information to adjust the weights of each channel. This process enhances the representation of globally significant features in the HRRP sequences, suppresses noise and less important features, and thereby strengthens the network’s expressive capability. The input HRRP data are first transformed using FrFT to enhance feature representation in the fractional Fourier domain. The FrFT of order α for a sequence χ(t) is defined as

(7) $$X_{a}(u)=F^{a}[\chi (t)](u)=\int_{-\infty }^{\infty }\;\chi (t)K_{a}(t,u)dt$$

where $K_{a}(t,u)$ is the transformation kernel:

(8) $$K_{a}(t,u)=\left\{\begin{array}{l} \sqrt{\frac{1-j\cot\alpha }{2\pi }}\exp\left(j\frac{t^{2}+u^{2}}{2}\cot\alpha -jut\csc\alpha \right),\\ \alpha \neq n\pi \\ \delta (t-u),\alpha =2n\pi \\ \delta (t+u),\alpha =(2n+1)\pi \end{array}\right.$$

Here, α=aπ/2 is the rotation angle in the distance-frequency plane. This kernel is the fundamental operator of the FrFT; varying the order α effectively rotates the signal representation in the joint distance-frequency plane, enabling the search for a domain where target-specific scattering characteristics are optimally concentrated and separated. The transformed data are then processed through the subsequent spatial feature extraction module. The calculation process for the spatial feature $O_{S}$ can be expressed as

(9) $$O_{S_{m}}=\left\{\begin{array}{c} SE\left(\mathrm{ReLU}\left(\mathrm{BN}\left(\mathrm{Conv}1D\left(X^{T}\right)\right)\right)\right),\;\;\;m=1\\ SE\left(\mathrm{ReLU}\left(\mathrm{BN}\left(\mathrm{Conv}1D\left(O_{S_{1}}\right)\right)\right)\right),\;\;\;m=2\\ ReLU\left(\mathrm{BN}\left(\mathrm{Conv}1D\left(O_{S_{2}}\right)\right)\right),\;\;\;m=3 \end{array}\right.$$

(10) $$O_{S}=\frac{1}{n}\sum_{i}^{n}O_{S_{3}}\left(i\right)$$

where $SE\left(\cdot \right)$ represents the squeeze-and-excitation block, $ReLU\left(\cdot \right)$ is the nonlinear activation function, $BN\left(\cdot \right)$ denotes the batch normalization layer, $Conv1D\left(\cdot \right)$ is the one-dimensional convolution layer, and n is the length of the HRRP sequence. The pooling layer uses adaptive average pooling to reduce the dimensionality of the convolution layer output, resulting in the global spatial feature $O_{S}$.

### 2.4. Decision Fusion and Recognition Module

**Figure 4:**
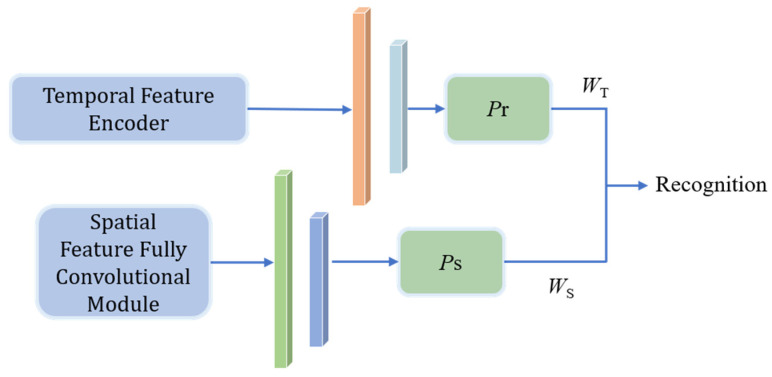
Decision fusion and recognition pipeline.

After extracting temporal correlation features and spatial structural features from the temporal feature encoder module and spatial feature fully convolutional module, respectively, the decision fusion and recognition module uses two fully connected layers to classify the temporal feature $O_{T}$ and spatial feature $O_{S}$, resulting in the temporal recognition result $P_{T}$ and the spatial recognition result $P_{S}$. Given the sensitivity of HRRP data to time shifts and target orientations, we establish learnable decision weight matrices $W_{T}$ and $W_{S}$, where $W_{T},\;W_{S}\in R^{1\times t}$, and t is the number of target categories. These matrices adaptively adjust the contributions of temporal and spatial features for each category, addressing time shift and orientation sensitivity issues to enhance model robustness. The calculation process for the final recognition result is described in Figure 4.

## 3. Loss Function Under Unbalanced Data

### 3.1. Focal Loss

Focal loss [34] is a loss function designed to address the issue of class imbalance. By introducing a coefficient factor on top of the standard cross-entropy loss, focal loss reduces the emphasis on easy samples and increases the focus on hard samples, thereby enhancing the model’s classification capability. In this work, hard samples primarily refer to training samples that are misclassified or classified with low confidence, often stemming from minority classes or challenging conditions. The calculation of focal loss is

(11) $$\mathrm{FL}=-\alpha_{t}{\left(1-p_{t}\right)}^{\gamma }\log\left(p_{t}\right)$$

where $p_{t}$ denotes the predicted probability of the true class for a sample, $\alpha_{t}$ is a balancing factor that adjusts the influence of positive and negative samples, and γ is a modulation factor that adjusts the focus on hard-to-classify samples. By tuning these weighting factors, focal loss effectively addresses the issue in which the loss value is dominated by the majority class and easy samples due to class imbalance.

### 3.2. Label-Smoothing Enhanced Focal Loss

Label smoothing is a regularization technique designed to prevent the model from becoming overly confident in its predictions on the training data, thereby improving generalization. The core idea of label smoothing is to convert the hard one-hot encoded vectors of true labels into a softer probability distribution. This method effectively reduces overfitting and enhances the model’s robustness to noisy data. Let y denote the true label and k be the number of classes. The one-hot encoding is $y_{i}$ (where only the *i*-th position is 1, and all others are 0) [35]. After label smoothing, $y_{i}^{\mathrm{smooth}}$ can be represented as

(12) $$y_{i}^{\mathrm{smooth}}=\left(1-\varepsilon \right)y_{i}+\frac{\varepsilon }{k}$$

where ε is the smoothing factor. Typically, label smoothing is combined with the cross-entropy loss (CE) [36], which is given by

(13) $${\mathrm{CE}}_{\mathrm{smooth}}=-\sum_{i=1}^{k}\left(\left(1-\varepsilon \right)y_{i}+\frac{\varepsilon }{k}\right)\log\left(p_{i}\right)$$

where $p_{i}$ denotes the model’s predicted probability for class  i. In the proposed method, label smoothing [37] is integrated with focal loss, resulting in the following calculation:

(14) $${\mathrm{FL}}_{\mathrm{smooth}}=-\sum_{i=1}^{k}\left(\left(1-\varepsilon \right)y_{i}+\frac{\varepsilon }{k}\right)\alpha_{i}{\left(1-p_{i}\right)}^{\gamma }\log\left(p_{i}\right)$$

Label smoothing provides a smoothed target distribution, making the loss function’s predictions more balanced across classes. Meanwhile, focal loss adjusts the loss weights to increase the model’s sensitivity to hard samples. The combination of these methods enhances both the model’s robustness to noisy data and its ability to recognize minority-class samples.

### 3.3. Adaptive Focal Loss

In standard focal loss, the balance factor α and the modulation factor γ are typically fixed values, and their settings significantly influence the model’s ability to recognize hard samples. In the proposed method, both the balance factor α and the modulation factor γ are dynamically adjusted based on the number of categories and prediction probabilities. The calculation formula for the balance factor $\alpha_{i}$ for each category is

(15) $$\alpha_{i}=\frac{N}{c_{i}}$$

where N is the total number of samples across all categories, and $c_{i}$ represents the number of samples in the *i*-th category. For minority classes, the balance factor α is larger, making the model pay more attention to the minority classes. The calculation process for the modulation factor $\gamma_{i}$ for the *i*-th class is

(16) $$\gamma_{i}={\;\gamma \;}_{0}+\beta (1-p_{i})$$

where $\gamma_{0}$ denotes the initial value of the dynamic adjustment factor, β is a tunable hyperparameter, and $p_{i}$ represents the predicted probability for the i-th class. For more difficult-to-classify samples, i.e., those with a smaller $p_{i}$, the modulation factor $\gamma_{i}$ will be larger, thereby giving more focus to these samples. Combining this with (12), the calculation formula for AFL-LS is

(17) $$\mathrm{AFL}-\mathrm{LS}=-\sum_{i=1}^{k}\left(\left(1-\varepsilon \right)y_{i}+\frac{\varepsilon }{k}\right)\frac{N}{c_{i}}{\left(1-p_{i}\right)}^{\gamma_{0}+\beta \left(1-p_{i}\right)}\log\left(p_{i}\right)$$

## 4. Results and Analysis

### 4.1. Datasets

In order to verify the validity of the proposed model and the loss function, we conducted experiments on the MSTAR dataset [38] and the CVDomes dataset [39]. The MSTAR dataset is widely used for radar automatic target recognition. It is sourced from a high-resolution synthetic aperture radar operating in the X-band with HH polarization and includes 10 target categories such as BMP2, BTR70, and T72.

We use data with a pitch angle of 17° as the training set and data with a pitch angle of 15° as the test set. We also include four variant targets—BMP2(SN-9563), BPM2(SN-C21), T72(SN-812), and T72(SN-S7)—in the test set to create Dataset 1, which tests the model’s generalization performance. Similarly, we use data with a pitch angle of 17° as the training set, but multiply the sample count of each category by a decay factor θ. For the reduced number of samples for the *i*-th category, we calculate it as

(18) $${\mathrm{Num}}_{i}^{\mathrm{Reduced}}={\;\theta \;}^{i}\times {\mathrm{Num}}_{i}$$

where ${\mathrm{Num}}_{i}$ denotes the original sample count of the *i*-th category, and i ranges from [0, 9]. In this experiment, θ is set to 0.6. We randomly select ${\mathrm{Num}}_{i}^{\mathrm{Reduced}}$ samples from the original dataset to construct a class-imbalanced training set. The test set remains the same, with data at a pitch angle of 15°, including four variant targets, which results in Dataset 2. Following the method in reference [40], we convert SAR images into HRRP sequences. The composition of the MSTAR HRRP sequence dataset is shown in Table 1 and Table 2.

The specific transformation steps are as follows: first, the SAR images undergo a one-dimensional inverse fast Fourier transform (IFFT) to obtain the complex domain HRRP sequences, which are then modulated to obtain the HRRP sequences. For each SAR image, 100 HRRP sequence samples are generated. By averaging every ten HRRP sequence samples, we obtain one averaged HRRP sample. Consequently, the training set of Dataset 1 contains 27,470 samples, while the training set of Dataset 2 contains 6809 samples. Both datasets have a test set containing 32,030 samples.

The CVDomes dataset, released in 2009, contains simulated X-band signatures of 10 civilian vehicle categories, including Toyota Camry, Honda Civic 4dr, and 1993 Jeep. Radar azimuth angles cover the full range from 0° to 359°. Acquired directly in HRRP format, this dataset requires no IFFT processing, and HRRP sequences can be generated through sliding window operations. For experimental validation, we select HH-polarized data at a 30-degree elevation angle and simulate varying signal-to-noise ratio conditions by superimposing Gaussian noise. Following the long-tail distribution strategy consistent with the MSTAR dataset, 60% of samples constitute the class-imbalanced training set, with 20% allocated to the validation and test sets, respectively. Detailed configuration parameters are provided in Table 3.

The imbalance factor (IF) [41] is a metric used to quantify the degree of class imbalance in classification problems. By calculating the IF, one can measure the disparity in the number of samples across different classes within a dataset. In this study, the IF values for Datasets 1–3 are measured at 0.997, 0.702, and 0.714, respectively. These metrics indicate that Dataset 1 maintains a near-ideal balanced state, whereas Datasets 2 and 3 exhibit significant class imbalance, manifesting characteristic long-tailed distribution patterns in sample category quantities.

### 4.2. Recognition Performance

To validate the effectiveness of the proposed method for HRRP sequence recognition, two types of comparative experiments are designed: one to verify the effectiveness of the LSTF model and the other to evaluate the AFL-LS loss function. The hardware environment for these experiments includes a 64-bit operating system, an Intel Core i5-13490F processor, an RTX 3060 GPU, and 32 GB of RAM. The software environment comprises Python 3.9 and PyTorch 2.0.1.

#### 4.2.1. Validation of the Effectiveness of the Proposed LSTF Model

To validate the effectiveness of the proposed method, we select LSTM-FCN [42], GRU-FCN [43], gMLP [44], XCM [45], GTN [46], RLAT [47], and LViT [48] as baseline methods for comparison. Each method runs five times under identical parameter conditions to minimize randomness caused by dropout and sample selection. LSTM-FCN and GRU-FCN are both hybrid models combining recurrent neural networks with convolutional neural networks. They leverage long short-term memory (LSTM) and gated recurrent units (GRU), respectively, to capture temporal dependencies, while integrating fully convolutional networks (FCN) to extract local spatial features, making them suitable for time-series data classification. gMLP centers on a multilayer perceptron, achieving feature modeling through gating mechanisms and spatial projections. It efficiently captures data correlations while dispensing with traditional attention mechanisms. XCM is an explainable convolutional neural network designed for multi-class temporal classification, leveraging convolutional structures to mine local data features while providing interpretability. GTN, or gated Transformer network, optimizes attention distribution in Transformers through gating mechanisms, enhancing the capture of critical information in multi-temporal data. RLAT is a lightweight Transformer designed for high-resolution distance image sequence recognition. It reduces parameter size and computational overhead through structural simplification, meeting edge device deployment requirements. LViT is a lightweight visual Transformer that employs optimized designs like competitive blocks to enhance feature extraction efficiency while maintaining compactness. It has been applied to visual tasks such as fingerprint recognition.

The experimental configuration employs a single-layer temporal feature encoder in the LSTF model, with group linear transformations (GLTs) divided into four groups. Preprocessed HRRP sequences of length 32 serve as the input, utilizing standard cross-entropy loss for Dataset 1 and AFL-LS for Datasets 2 and 3.

On class-balanced Dataset 1, all comparative models demonstrate competent baseline performance. However, the proposed LSTF exhibits significant superiority in both recognition accuracy and generalization capability. As detailed in Table 4, LSTF achieves 99.52% recognition accuracy in the 10-class identification task, outperforming the suboptimal model by 0.25 percentage points while maintaining the highest accuracy across all target categories. Notably, the model attains 99.52%, validating the spatiotemporal fusion mechanism’s sensitivity to subtle target differences. Except for the methods proposed for certain datasets (2S1, BTR70, and T72), which are not optimal, the proposed methods achieve the best recognition performance across all other datasets. These results confirm that LSTF maintains high precision while demonstrating exceptional generalization capabilities, establishing a technical foundation for subsequent research on class-imbalanced data conditions.

**Figure 5:**
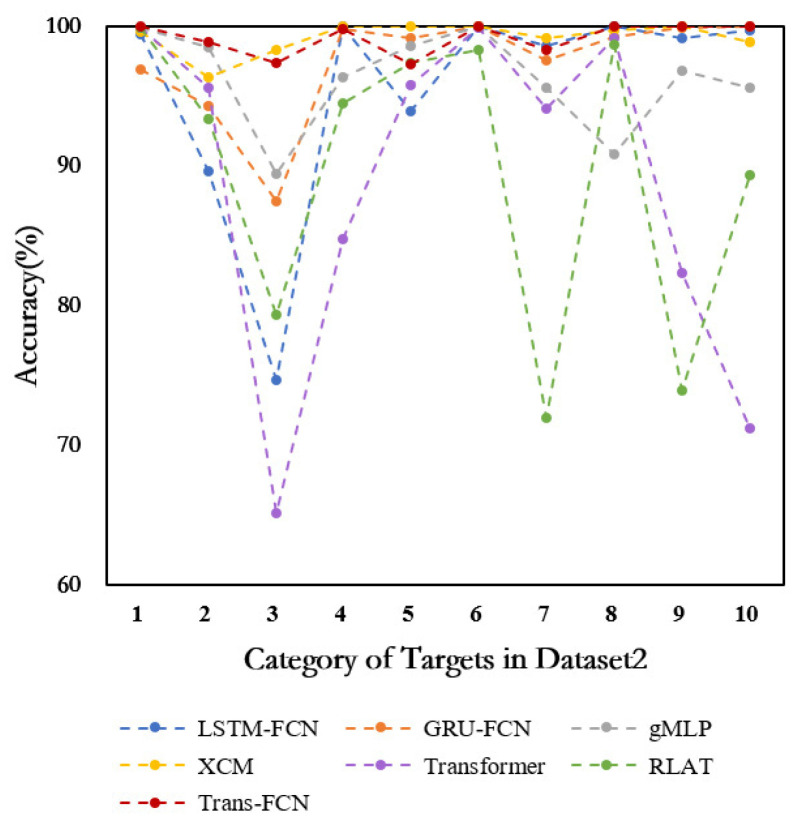
Accuracy of the 10 targets in Dataset 2.

Further comparative analysis on class-imbalanced Dataset 2 reveals the recognition accuracy of competing models, as quantitatively detailed in Table 5. The LSTF model maintains superior overall recognition rates while achieving optimal performance across four target categories. Given Dataset 2’s imbalanced training distribution and test set containing variant targets, the experimental setup imposes heightened demands on model generalization capabilities. The results demonstrate that LSTF attains 99.96% and 99.98% accuracy for BRDM-2 and T62 categories with variant models, respectively, confirming the effectiveness of the dynamic decision weighting mechanism in mitigating feature confusion. For tail classes 2S1, BTR70, D7, T72, and ZIL131, the model achieves 100.00% accuracy, showing marked improvements over Transformer-based GTN and RLAT models. In challenging BMP2, BTR60, and ZSU23/4 category recognition, the performance of LSTF is slightly inferior. Furthermore, as illustrated in Figure 5, LSTF exhibits enhanced recognition stability on Dataset 2 with a maximum fluctuation limited to 0.61%, demonstrating significant advantages over comparative models.

To systematically evaluate model performance under class-imbalanced conditions, the experiment incorporates four metrics: macro F1-score, G-mean, macro ROC-AUC, and macro PR-AUC for comprehensive analysis, with quantitative results presented in Table 6. LSTF demonstrates superior performance across the four metrics: achieving a macro F1-score of 0.9935 indicates balanced inter-class prediction capabilities; its G-mean value significantly outperforms other baselines, validating strong robustness against skewed data distributions; both macro ROC-AUC and PR-AUC approach theoretical maximums, confirming the model’s decision reliability under high-confidence thresholds.

To validate the model’s recognition robustness and noise immunity under class-imbalanced and low signal-to-noise ratio (SNR) conditions, comparative experiments were conducted on the class-imbalanced Dataset 3. Gaussian noise was introduced during data preprocessing to construct training sets with an SNR of 20 dB, with model performance evaluated under test conditions of 20 dB, 15 dB, 10 dB, and 5 dB SNR levels, as detailed in Table 7. Experimental results reveal differentiated performance degradation patterns across models as SNR decreases. Under test conditions of 15 dB, the proposed model achieved recognition accuracy second only to the parameter-intensive GTN model, while demonstrating optimal performance under other SNR conditions. This indicates that the proposed method effectively overcomes existing models’ reliance on high-quality signals, exhibiting enhanced engineering applicability in complex battlefield environments with dynamically varying SNR levels.

This study further compares parameter counts and computational complexity across models to evaluate engineering applicability, as shown in Table 8. Among Transformer-based variants, GTN demonstrates superior performance on noise-contaminated Dataset 3, where its deep architecture and high parameter count facilitate complex feature extraction, yet its large model size hinders real-time processing on embedded devices. In contrast, LSTF achieves significant reductions in both parameters and computations compared to GTN, while maintaining deep feature extraction capabilities through lightweight modifications. Although the most lightweight model, RLAT, excels on class-balanced Dataset 1, its recognition accuracy declines on imbalanced Datasets 2 and 3, revealing insufficient stability in identifying minority-class samples. While the proposed model’s lightweight characteristics remain inferior to baseline methods like LSTM-FCN and GRU-FCN, it effectively reduces the inherent high parameter and computational loads of the Transformer attention mechanism. This optimization achieves complexity reduction without compromising recognition performance, making Transformer-based architectures more suitable for deployment on edge devices.

**Figure 6:**
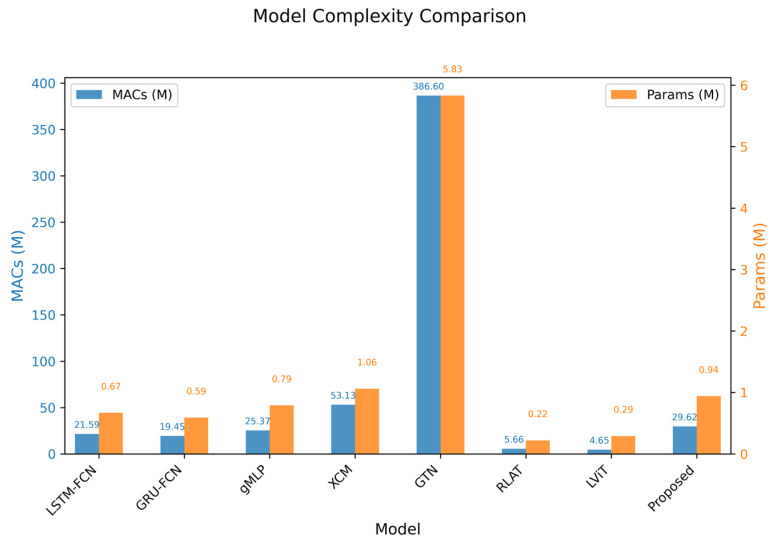
Lightweight comparison of each model.

For a more intuitive comparison, Figure 6 is plotted. This dual-axis bar chart clearly compares the performance trade-offs of eight temporal classification models (including the proposed model) in terms of computational complexity (MACs, left blue axis) and model size (Parameters, right red axis). It reveals that while the GTN model achieves significantly higher values than other models across both metrics, most other models—including the proposed model—exhibit MACs and parameters concentrated within a lower range, demonstrating efficiency while maintaining relatively low resource consumption.

#### 4.2.2. Verification of AFL-LS Effectiveness

To verify the effectiveness of the proposed AFL-LS, several comparison methods are selected: standard cross-entropy loss (CE), data distribution loss (DD Loss) [49], standard focal loss (FL), focal loss with label smoothing (FL-LS), and adaptive focal loss (AFL). These methods are evaluated using the LSTF model on Dataset 2. The smoothing factor ε is set to 0.2, while $\gamma_{0}$ and β are set to 2.0. Each method is run five times, and the average recognition rates for each method are shown in Table 9.

Experimental results demonstrate that the proposed AFL-LS loss function exhibits significant performance advantages under class-imbalanced conditions. It achieves superior overall accuracy compared to all baseline methods, outperforming the second-best approach, CE, by 0.3 percentage points. For the severely imbalanced ZSU23/4 and BMP2 datasets, AFL-LS achieved recognition rates of 99.48% and 95.97%, respectively. Meanwhile, CE effectively guided model attention toward minority-class samples, yielding high recognition rates. For BRDM-2 and BTR60, although label smoothing strategies enhanced model generalization, they simultaneously increased recognition difficulty for minority classes, causing CE-LS and FL-LS to completely fail in these categories. For the remaining targets, AFL-LS maintained higher accuracy than FL, confirming LS’s effectiveness in improving majority class generalization. Notably, on the BRDM-2 target with complex scattering patterns, the state-of-the-art DD loss achieved only a 98.83% recognition rate. AFL-LS, however, elevated performance to 99.96% through dynamic class weight balancing. By integrating adaptive mechanisms with label smoothing techniques, this method not only intensifies focus on minority classes but also enhances generalization capabilities for variant targets, ultimately achieving the highest overall average recognition rate.

Furthermore, comparative analysis was conducted using four metrics: macro-F1 score, G-mean, macro ROC-AUC, and macro PR-AUC, as shown in Table 10. The proposed AFL-LS method achieves optimal performance across all metrics, while CE, CE-LS, and FL-LS completely fail on minority classes, resulting in G-mean values of zero and lower macro-F1 scores. Although the state-of-the-art DD Loss demonstrates outstanding performance in the macro ROC-AUC and macro PR-AUC metrics, its values still fell short of those of the algorithm proposed in this paper.

Additionally, comparative experiments with different loss functions were performed on Dataset 3 under low signal-to-noise ratio (SNR=5 dB) conditions, as shown in Table 11 and Table 12. AFL-LS attained the highest overall recognition accuracy of 96.03%, outperforming the second-best method, AFL, by 4.19%, and achieved the highest recognition rates in 8 out of 10 target classes. Analysis of other evaluation metrics demonstrates that AFL-LS exhibits marked superiority in macro-F1 score and G-mean, confirming its ability to balance recognition performance between majority and tail classes. Furthermore, its exceptional performance in macro ROC-AUC and macro PR-AUC reflects high-confidence decision boundaries in noisy environments, proving that this loss function significantly enhances model robustness and noise resistance under low-SNR conditions.

### 4.3. Ablation Experiment

To validate the effectiveness of the spatiotemporal feature complementary mechanism and adaptive weighting decision module, ablation studies were conducted on the MSTAR and CVDomes datasets, as presented in Table 13. Single-branch features exhibited significant performance fluctuations across data conditions. The spatial branch achieved 3.78% and 25.72% higher accuracy than the temporal branch in Datasets 1 and 2, whereas in Dataset 3, the spatial branch declined to 87.13%, while the temporal branch maintained robust performance at 94.13%. This demonstrates the differentiated sensitivity of spatiotemporal features to environmental characteristics: spatial features excel in geometric configuration analysis, while temporal features exhibit stronger noise robustness. Although the dual-branch framework enhances model applicability, static equal-weight fusion strategies tend to suffer from limited optimization and feature quality instability. The proposed adaptive weighting mechanism achieved performance improvements of 0.27%, 0.96%, and 1.62% across the three datasets.

The experimental results indicate that the coordinated design of the dual-branch framework and adaptive weighting mechanism effectively addresses feature modality conflicts and scene adaptability limitations in conventional methods, demonstrating superior generalization capability in complex data distributions and noisy environments.

### 4.4. Complexity Analysis

The primary computational cost of the proposed method comes from the Transformer_FCN backbone. Let the input sequence length be TS, the model dimension be d, the feed-forward hidden dimension be $d_{ff}$, and the number of layers be L.

Then, the computational cost of a single forward pass is approximately

(19) $$O\left(L\left({\left(TS\right)}^{2}d+TS\cdot d\cdot d_{ff}\right)\right)$$

Considering that backpropagation takes approximately 2–3 times the cost of forward propagation, the training complexity per epoch is

(20) $$O\left(\frac{N}{B}\cdot 3L\left({\left(TS\right)}^{2}d+TS\cdot d\cdot d_{ff}\right)\right)$$

The space complexity is primarily determined by the parameters and the attention map:

(21) $$O\left(L\left(d^{2}+dd_{ff}\right)+BTSd+B{\left(TS\right)}^{2}\right)$$

Compared to other traditional convolutional models, this approach exhibits slightly higher computational complexity when the temporal–spatial dimension TS is large, but demonstrates significant advantages in feature representation and classification accuracy.

Notably, the proposed method achieves real-time inference performance on resource-limited edge devices. Benchmark tests on an Intel Core i5-13490F processor show that the inference latency for a single HRRP sequence (length = 32) is only 2.3 ms, which is far below the 10 ms real-time threshold required for radar target recognition systems. This real-time capability is attributed to the TGLT-based lightweight design, which reduces computational complexity by 92.4% compared to the standard Transformer encoder (GTN), enabling seamless deployment in time-sensitive scenarios.

### 4.5. Visualization and Evaluation of Feature Extraction

**Figure 7:**
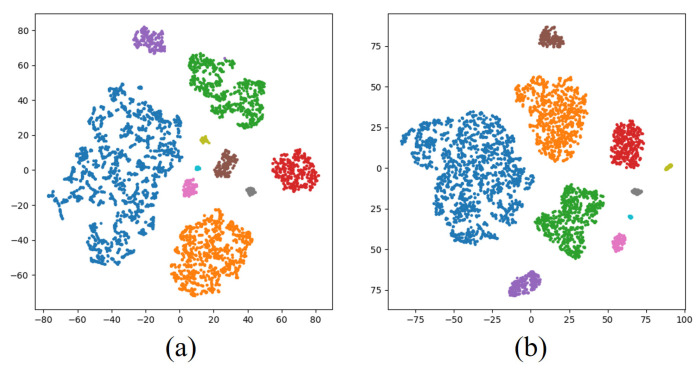
Visualization results of feature extraction on Dataset 2. (**a**) Temporal features. (**b**) Spatial features.

**Figure 8:**
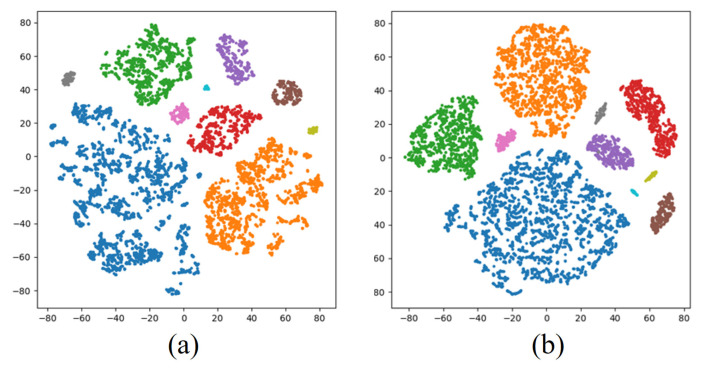
Visualization results of feature extraction on Dataset 3. (**a**) Temporal features. (**b**) Spatial features.

To validate the effectiveness of spatiotemporal feature extraction in the proposed model, t-distributed stochastic neighbor embedding (t-SNE) was employed to visualize features from the imbalanced Dataset 2 and Dataset 3, as shown in Figure 7 and Figure 8. t-SNE projects high-dimensional features into a 2D space (with axes t-SNE Dimension 1 and t-SNE Dimension 2), enabling intuitive observation of feature distributions and clustering patterns. Specifically, Figure 7a and Figure 8a display temporal correlation features of HRRP sequences extracted by the temporal encoder, while Figure 7b and Figure 8b illustrate spatial structural features obtained from the full-convolution spatial module. In all subfigures, each point represents a sample, and its color corresponds to a specific target class (see legend).

The visualization results reveal that both temporal and spatial features exhibit distinct inter-class separability and intra-class compactness after dimensionality reduction (i.e., points of the same color form tight clusters, and clusters of different colors are well separated), confirming the dual-branch architecture’s capability to characterize target scattering mechanisms. This visualization provides interpretable evidence for the model’s robustness in complex electromagnetic environments, demonstrating the theoretical superiority of the spatiotemporal feature complementary enhancement mechanism.

## 5. Conclusions

In this study, we proposed an LSTF method for HRRP sequence target recognition to address the challenges of category imbalance and robustness under complex conditions. The designed TGLT-based lightweight Transformer encoder effectively captured temporal dynamics while reducing computational overhead, which satisfied the requirements of real-time edge deployment. The transform-domain spatial feature extraction network, incorporating the fractional Fourier transform and FSCN, fully exploited multi-domain spatial information and enhanced feature discriminability. Furthermore, the AFL-LS improved recognition for long-tail classes and strengthened generalization capability. Experimental results on the MSTAR and CVDomes datasets showed that our method outperformed existing baselines in multiple scenarios, especially in minority class recognition and variant identification, while maintaining stable robustness under noise interference. Overall, the proposed approach significantly improved recognition performance and demonstrated strong adaptability for practical non-cooperative target recognition.

Future research can further extend this work in several directions. First, more effective recognition strategies for extremely short, incomplete, or missing HRRP sequences should be explored to better match realistic non-cooperative radar scenarios. Second, self-supervised and semi-supervised learning can be introduced to reduce reliance on labeled data and improve generalization for long-tail and unseen target categories. Third, adaptive long-tail optimization and incremental learning mechanisms may be developed to handle dynamically changing class distributions and emerging targets. In addition, multi-view and multi-modal fusion of HRRP with complementary radar representations can be investigated to enhance robustness under complex observation conditions. Incorporating physics-informed priors into data-driven models is also a promising direction to improve interpretability and domain generalization. Finally, further hardware-aware optimization and model compression should be studied to support efficient, low-latency deployment on real-time edge radar platforms.

## Figures and Tables

**Table 1:** MSTAR HRRP Sequence Dataset 1.

Target	Training Set (17°)	Target	Testing Set (15°)
2S1	2990	2S1	2740
BMP2(SN-9566)	2330	BMP2(SN-9566)	1960
		BMP2(SN-9563)	1950
		BMP2(SN-C21)	1960
BRDM-2	2980	BRDM-2	2740
BTR70(SN-C71)	2330	BTR70(SN-C71)	1960
BTR60	2560	BTR60	1950
D7	2990	D7	2740
T62	2990	T62	2730
T72(SN-132)	2320	T72(SN-132)	1960
		T72(SN-812)	1950
		T72(SN-S7)	1910
ZIL131	2990	ZIL131	2740
ZSU23/4	2990	ZSU23/4	2740
Total	27,470	Total	32,030

**Table 2:** MSTAR HRRP Sequence Dataset 2.

Target	Training Set (17°)	Target	Testing Set (15°)
2S1	2990	2S1	2740
BMP2(SN-9566)	1398	BMP2(SN-9566)	1960
		BMP2(SN-9563)	1950
		BMP2(SN-C21)	1960
BRDM-2	1072	BRDM-2	2740
BTR70(SN-C71)	503	BTR70(SN-C71)	1960
BTR60	331	BTR60	1950
D7	232	D7	2740
T62	139	T62	2730
T72(SN-132)	64	T72(SN-132)	1960
		T72(SN-812)	1950
		T72(SN-S7)	1910
ZIL131	50	ZIL131	2740
ZSU23/4	30	ZSU23/4	2740
Total	6809	Total	32,030

**Table 3:** CVDomes HRRP Sequence Dataset 3.

Target	Training Set	Testing Set
Toyota Camry	3648	1200
Honda Civic 4dr	2188	1200
1993 Jeep	1313	1200
1999 Jeep	787	1200
Nissan Maxima	472	1200
Mazda MPV	283	1200
Mitsubishi	170	1200
Nissan Sentra	102	1200
Toyota Avalon	61	1200
Toyota Tacoma	36	1200
Total	9060	12,000

**Table 4:** Recognition accuracy of comparative experiments on Dataset 1 (%).

	LSTM-FCN	GRU-FCN	gMLP	XCM	GTN	RLAT	LViT	Proposed
2S1	100.00	100.00	100.00	99.87	97.91	99.97	99.34	98.47
BMP2	96.94	98.31	91.22	77.59	93.32	99.01	99.08	99.91
BRDM-2	96.47	95.54	92.22	84.42	68.24	92.06	98.50	99.01
BTR70(SN-C71)	100.00	100.00	86.37	98.38	89.07	99.32	94.26	97.60
BTR60	99.09	99.82	96.31	82.99	99.01	98.36	96.62	99.24
D7	100.00	100.00	100.00	99.98	98.25	100.00	100.00	100.00
T62	99.91	99.67	98.83	91.27	93.81	97.46	100.00	100.00
T72	100.00	100.00	94.98	91.62	99.36	99.99	97.71	99.79
ZIL131	100.00	100.00	99.27	95.41	98.54	99.94	100.00	100.00
ZSU23/4	100.00	100.00	99.76	99.97	99.67	99.97	100.00	100.00
Overall Accuracy	99.07	99.27	95.56	90.73	94.22	98.77	98.65	99.52

**Table 5:** Recognition accuracy of comparative experiments on Dataset 2 (%).

	LSTM-FCN	GRU-FCN	gMLP	XCM	GTN	RLAT	LViT	Proposed
2S1	99.43	96.93	99.92	99.61	99.94	99.88	99.78	100.00
BMP2	89.60	94.30	98.54	96.38	95.58	93.37	89.85	95.97
BRDM-2	74.65	87.49	89.41	98.32	65.11	79.36	88.91	99.96
BTR70(SN-C71)	100.00	99.81	96.33	100.00	84.74	94.47	81.44	100.00
BTR60	93.90	99.16	98.55	99.94	95.81	97.42	81.52	99.95
D7	100.00	100.00	99.96	100.00	99.93	98.32	99.59	100.00
T62	98.61	97.60	95.62	99.19	94.14	71.97	99.26	99.98
T72	99.97	99.28	90.87	99.71	99.16	98.67	99.62	100.00
ZIL131	99.19	99.94	96.81	100.00	82.33	73.90	99.85	100.00
ZSU23/4	99.71	99.99	95.60	98.86	71.19	89.31	99.63	99.68
Overall Accuracy	95.29	97.22	95.82	98.94	90.41	90.62	94.37	99.55

**Table 6:** Performance comparison of each model on other evaluation indicators on Dataset 2 (%).

	LSTM-FCN	GRU-FCN	gMLP	XCM	GTN	RLAT	LViT	Proposed
Macro F1	0.9467	0.9722	0.9574	0.9894	0.8804	0.8764	94.27	0.9935
G-mean	0.9457	0.9711	0.9598	0.9920	0.8760	0.8723	94.64	0.9953
Macro ROC-AUC	0.9990	0.9718	0.9981	0.9999	0.9882	0.9878	99.71	1.0000
Macro PR-AUC	0.9949	0.9998	0.9908	0.9993	0.9441	0.9454	98.01	1.0000

**Table 7:** Recognition accuracy of each model under different SNR levels on Dataset 3 (%).

SNR(dB)	LSTM-FCN	GRU-FCN	gMLP	XCM	GTN	RLAT	LViT	Proposed
20	81.16	85.18	96.53	82.26	97.53	88.00	80.14	98.98
15	78.68	84.07	93.67	77.88	94.67	85.89	72.05	93.50
10	72.23	74.40	85.68	71.74	89.21	81.95	47.64	89.23
5	60.21	63.17	66.77	58.13	79.83	72.88	33.98	79.96

**Table 8:** Lightweight comparison of each model.

	LSTM-FCN	GRU-FCN	gMLP	XCM	GTN	RLAT	LViT	Proposed
Macs(M)	21.59	19.45	25.37	53.13	386.60	5.66	4.65	29.62
Params(M)	0.67	0.59	0.79	1.06	5.83	0.22	0.29	0.94

**Table 9:** Recognition accuracy of each loss function on Dataset 2 (%).

	CE	CE-LS	DD Loss	FL	FL-LS	AFL	LV-LS	AFL-LS
2S1	100.00	99.12	99.85	86.04	100.00	100.00	85.97	100.00
BMP2	100.00	100.00	99.87	100.00	96.47	99.86	99.94	95.97
BRDM-2	96.46	95.85	98.83	96.11	98.91	99.92	96.05	99.96
BTR70(SN-C71)	93.61	99.69	89.33	72.14	91.81	98.29	72.91	100.00
BTR60	99.84	99.64	76.59	95.21	98.43	99.94	93.18	99.95
D7	97.72	98.58	100.00	99.93	100.00	89.02	97.88	100.00
T62	98.36	84.67	98.11	95.07	99.12	100.00	85.41	100.00
T72	99.95	99.15	97.45	98.19	100.00	99.91	95.12	100.00
ZIL131	100.00	91.00	99.89	99.52	100.00	100.00	98.25	100.00
ZSU23/4	100.00	90.00	100.00	100.00	100.00	100.00	99.46	99.48
Overall Accuracy	98.91	95.77	96.62	94.77	98.47	98.69	92.41	99.21

**Table 10:** Comparison of the performance of each loss function on other evaluation indicators (Dataset 2).

	CE	CE-LS	DD Loss	FL	FL-LS	AFL	LViT	AFL-LS
Macro F1	0.9874	0.8615	0.9628	0.9516	0.9850	0.9860	0.948	0.9935
G-mean	0.9893	0.0000	0.9697	0.9671	0.9848	0.9856	0.9021	0.9953
Macro ROC-AUC	1.0000	0.9972	0.9996	1.0000	0.9999	0.9999	0.9865	1.0000
Macro PR-AUC	1.0000	0.9764	0.9973	1.0000	0.9993	0.9994	0.9798	1.0000

**Table 11:** Recognition accuracy of each loss function on Dataset 3 (%).

	CE	CE-LS	DD Loss	FL	FL-LS	AFL	LV-LS	AFL-LS
Toyota Camry	97.17	89.54	92.79	87.62	85.80	87.08	86.52	98.28
Honda Civic 4dr	87.40	70.18	79.79	92.95	94.56	94.86	82.56	96.53
1993 Jeep	100.00	87.15	98.07	91.79	83.05	98.57	97.95	98.09
1999 Jeep	65.63	89.11	91.20	91.19	79.67	80.68	88.25	90.22
Nissan Maxima	96.73	95.10	98.94	98.94	89.32	93.13	92.47	98.29
Mazda MPV	96.15	66.39	99.83	100.00	96.74	95.09	94.36	97.24
Mitsubishi	70.41	76.04	89.98	76.24	82.37	85.41	86.78	88.17
Nissan Sentra	100.00	98.03	92.47	90.34	96.31	96.44	97.72	98.06
Toyota Avalon	98.42	88.04	71.33	100.00	99.37	96.76	96.08	98.49
Toyota Tacoma	95.77	0.00	92.69	99.59	100.00	97.40	95.48	96.88
Overall Accuracy	88.80	82.47	89.53	91.83	89.38	91.84	91.82	96.03

**Table 12:** Comparison of the performance of each loss function on other evaluation indicators (Dataset 3).

	CE	CE-LS	DD Loss	FL	FL-LS	AFL	LV-LS	AFL-LS
Macro F1	0.8640	0.7822	0.8867	0.9154	0.8806	0.9138	0.9112	0.9364
G-mean	0.8237	0.0000	0.8696	0.9092	0.8598	0.9066	0.9027	0.9319
Macro ROC-AUC	0.9956	0.9930	0.9942	0.9953	0.9961	0.9958	0.9923	0.9986
Macro PR-AUC	0.9717	0.9556	0.9644	0.9807	0.9753	0.9769	0.9635	0.9893

**Table 13:** Recognition accuracy of ablation experiments.

Temporal Branch	Spatial Branch	Adaptive Weighting	Accuracy (%)		
			Dataset 1	Dataset 2	Dataset 3
√			94.94	73.36	94.13
	√		98.72	99.08	87.13
√	√		99.40	98.28	95.34
√	√	√	99.67	99.24	96.96

## Data Availability

The original contributions presented in this study are included in the article. Further inquiries can be directed to the corresponding author.
